# Editorial: Tumor microenvironment signaling networks in pathophysiology and therapeutics

**DOI:** 10.3389/fonc.2022.1009187

**Published:** 2022-09-08

**Authors:** Francesca Pirini, Daniele Vergara, Paola Parrella

**Affiliations:** ^1^ Biosciences Laboratory, IRCCS Istituto Romagnolo per lo Studio dei Tumori (IRST) “Dino Amadori”, Meldola, Italy; ^2^ Department of Biological and Environmental Sciences and Technologies, University of Salento (DiSTeBA), Lecce, Italy; ^3^ Laboratory of Oncology, IRCCS ’Casa Sollievo della Sofferenza’, San Giovanni Rotondo, Italy

The tumor microenvironment (TME) describes a variety of resident and infiltrating host non-cancerous cells including stromal cells, fibroblasts, endothelial cells, innate and adaptive immune cells, vessels, nerves and microbiota. The interplay of these different populations is the goal of numerous researches as it plays a fundamental role in tumor pathophysiology and response to treatments. Inside the tumor mass, the communication between cancer cells and the microenvironment is influenced by an intricate network modulated in time and space *via* metabolites, hormones, proteins and other molecules that influence crucial cellular processes including cell proliferation, apoptosis, cellular metabolism, genetic instability, angiogenesis, and metastasis promotion. Therefore, understanding the role of TME components and their integration adds important elements to the development and improvement of therapies. This Research Topic presents a collection of review and research articles that shed light on the molecular pathways and cellular processes involved in TME-cancer crosstalk at the signaling level, and how they influence cancer progression and response to treatments.

Ten papers of this collection focus specifically on the role of specific TME cell types in the regulation of cancer promotion and progression. As described, a range of well-orchestrated signaling mechanisms is required to coordinate their activities. In this scenario, the application of innovative techniques and increased computational power brings to a fine classification of the TME components.

Wei et al. investigated the intercellular interactions triggered by a hypoxic TME, identifying the cell population that plays a key prognostic role and the specific ligand – receptor pairs involved in tumorigenesis regulation. The authors performed a comprehensive analysis of single cell transcriptomic data collected on pan-cancer TME blueprint of six cancer types. They identified a specific subtype of macrophages characterized by the presence of secreted phosphoprotein 1 (SPP1) receptor (SPP1+ TAMs) and co-expression of MMP9. The SPP1+ TAMs are also strongly associated with hypoxia, and SPP1 expression is upregulated under this condition. Because of the expression of MM9, they are associated with epithelial mesenchymal transition (EMT), with glycolysis and with worse outcomes. This study lays the foundations for a greater knowledge of the interactions between the components of TME and suggests possible new markers useful for improving patient stratification and the development of therapies.

Zhang Z. et al. reviewed the functional role of pancreatic stellate cells (PSC) in the progression of pancreatic cancer. PCS are stromal cells exclusively of the pancreas which function is to store vitamin A, lipid droplets and express protein markers such as synemin and desmin (1). PCSs are usually in a quiescent state but can be activated and recruited by pancreatic tumor cells. Recently, two subtypes of PCS have been identified, the myofibroblastic and inflammatory PCS subtypes, that are functionally complementary and cooperate to a favorable microenvironment for cancer survival.

Another TME component that has sparked attention for its role in supporting the proliferation of prostate cancer cells are osteoblasts. Ribelli et al. contributed to elucidate the signaling network that determines the interaction between castration resistant prostate cancer (CRPC) and osteoblast using *in vitro* co-colture models. This team reported a significant reduction of the expression of androgen receptor (AR) mRNA, protein and function after culturing C4-2B cell line with osteoblast-conditioned media (OCM), but an increase in proliferation. Among the soluble factors found in the OCM with a potential role in the reduction of AR, they found the matrix metalloproteinase-1 (MMP-1) protein. These results suggest that MMP-1 reduces AR expression and enhances proliferation binding PAR-1, a G protein-coupled receptor (GPCR). Thus, MMP-1/PAR-1 could be one of the potential pathways able to promote AR-independent CRPC proliferation.

It is now evident a direct role of nerves in the regulation of tumorigenesis. In fact, recent studies have demonstrated that nerves support tumor progression and dissemination, and that a specialized niche might be established between cancer cells and nerves. Specific signaling molecules including neurotrophins, neurotransmitters, adhesion molecules, matrix metalloproteinase and other mediators are highly enriched in this niche, suggesting that neoplastic invasion of nerves might be a key hallmark of cancer. Even if the perineural invasion (PNI) does not appear to be a common feature of colorectal cancer (CRC), PNI-positive CRC patients show a more than 20% decrease in overall and disease-free survival. As suggested by Zhang L. et al. nerves may stimulate tumor growth by releasing neurotransmitters and activating multiple downstream pathways leading to a higher risk of lymph node metastasis. An important mechanism of communication involved in both tumor promotion and tumor suppression, is the erythropoietin-producing hepatocellular carcinoma (Eph) receptor B2 (EphB2) pathway. EphB2 is a transmembrane receptor expressed in tumor cells and endothelial cells that binds transmembrane-ephrin ligands therefore them activation requires cell-cell contact, leading to a bidirectional intracellular signaling that activates various molecules, such as MAP kinases, Src family kinases, GTPases, PI3K and phosphatases. EphB2 can generate bidirectional signals and is aberrantly expressed in many cancer types. In tumors where EphB2 is overexpressed it acts as tumor promoter (hepatocarcinoma, breast cancer, glioma and malignant mesothelioma) while in colorectal cancer and bladder cancer is low expressed indicating a tumor suppression role. Recently, its expression has been detected also in immunocytes and monocytes reporting a role in immunity. Liu et al. summarized the role of EphB2 in cancer and the potential use as biomarker for cancer diagnosis, prognosis and treatment.

In addition to these different cellular types, other factors can impact on TME. For instance, chronic stress but also more physical and mechanical characteristics can activate signaling pathways that stimulate tumor initiation and progression. In particular, two articles deal with these topics. Sheth and Esfandiari discusses the fact that cancer is characterized by a bioelectric dysregulation which can be explained by the change in electrical state of the membrane potential and in an altered extracellular vesicles production which in turn can alter the tissue organization. Membrane potential is a property belonging to all cells and an integral contributor of the microenvironment that guides cellular behavior (2) spatially and temporally. The role of bioelectric dysregulation in cell signaling and its influence in EV production needs to be studied in depth for a more complete view of the mechanisms that regulate tumor initiation and metastasis and because it is manipulable thanks to the application of new technologies, as explained by the authors.

Some organs are subjected to continuous movements. The cells of the lungs and in particular lung cancer associated fibroblasts (CAFs) are constantly subjected to stretching and retraction because of the movements associated with breathing and this mechanical load plays a role in tumorigenesis. (3, 4). It is not to be forgotten that cells respond also to mechanical signals *via* mechanoreceptors that often encounter the ECM where the signals are converted in physiological responses. These signals affect cell proliferation, differentiation, and migration (5). Mechanical stimulation can regulate fibroblasts and the ECM components within the tumor microenvironment and studies suggest that CAFs play a crucial role in tumor progression. This topic has been reviewed by Gong et al. that provided an overview of factors involved in cell mechanics with a role in tumorigenesis.

Chronic stress occurs frequently in cancer patients during cancer diagnosis and treatment (6) and extensive studies determined the influence of these factors in altering TME. As reviewed by Tian et al., chronic stress leads to a constant release of stress hormones due to a constant activation of hypothalamic-pituitary adrenal (HPA) axis and the sympathetic nervous system (SNS). In detail, the TME of patients with chronic stress is characterized by changes in the number and types of immune cells, such as an increase of macrophages and NK. Chronic stress also affects the type and quantity of cytokines, angiogenesis, enhanced epithelial mesenchymal transition (EMT), and damaged ECM.

The review by Tian et al. also summarizes the mechanisms that lead to TME changes, under chronic stress. Mechanistically, androgen receptor (AR) and glucocorticoid signalling can modulate TME stress in distinct ways, for instance by inducing hypoxia (7). These findings suggest that the integration of cancer therapies with α-blockers, β-blockers, antidepressants, and interventions, like meditation and mindfulness, may be introduced in treatment plans to improve response to therapies.

A precise TME characterization also allows the identification of personalized strategies to improve cancer prognosis. Authophagy is an important doble player in tumorigenesis, acting in a positive and negative way, and is also associated with immunity. Zhang M.Y. et al. through an analysis of the gene expression profile and clinical information of 594 lung samples (LUAD), establish a risk model based on 10 autophagy related genes (ARGs) to predict the prognosis of LUAD. They also used five pooled ARG expression signatures as independent prognostic factors.

In recent years, the introduction of immune checkpoint inhibitors (ICIs) have revolutionized the treatment of solid tumors. However not all patients benefit from the treatment, and secondary resistance is widely reported. It is well known that *in vitro*, the immune system can recognize tumor antigens and kill tumor cells, but the recognition of the tumor antigen alone is not sufficient for the host to eliminate an established tumor *in vivo*. Indeed, the TME usually prevents effective lymphocyte priming, and tumor infiltration, and suppresses effector cells, which leads to a failure of the host to control tumor growth. The predominance of specific cell phenotypes in the TME may exert pro- or anti-tumoral and their modulation can affect the responses to treatments, making them more or less effective. In this collection, Russo and Nastasi reported the latest knowledge about the role of tumour-associated macrophages and neutrophils, their interactions with tumor cells, their role in response to current treatments and the development of therapeutic strategies.

The recognition of tumour cells by the immune system depends on a delicate balance between activating and inhibitory signals mediated by specific receptors. In particular the T cell immunoreceptor with immunoglobulin and ITIM domain (TIGIT) is an inhibitory receptor that regulates T cell-mediated tumor recognition and represents a putative target for checkpoint blockade immunotherapy. Annese et al. discuss the latest development on the role of TIGIT in cancer progression as possible therapeutic strategies to avoid tumor progression, drug resistance, and drug safety.

Three studies of the collection address the relationship among TME markers and response to immunotherapy in NSCLC. Wenhao Oyung by using bioinformatic and algorithms, established a risk model for overall survival based on hypoxia, immune, and EMT gene signatures. The model was established by using the TCGA-Lung Adenocarcinoma dataset (8) as training cohort and the Gene Expression Omnibus (GEO) database (GSE68465, GSE72094) (9) as validation cohorts. Overall survival differed significantly between the high-risk and low-risk groups with AUCs for predicting 1-, 3-, and 5-year survival of 0.763, 0.766, and 0.728 on the three datasets. In the TCGA dataset, the alterations in immune checkpoint genes, and the TME markers immunoscore and stromal score (10), were negatively correlated with the risk score indicating stronger tumor immune activity in low-risk patients than in high-risk patients. In addition, the risk score formula was associated with progression-free survival (PFS) in patients with NSCLC undergoing anti-PD-1/PD-L1 therapy (GSE135222 dataset) and was higher in patients with NSCLC who had experienced no benefit from nivolumab or pembrolizumab than in those who had experienced a benefit (GSE126044 dataset). Interestingly, the risk score was associated with worse immunotherapy response in patients with metastatic urothelial cancer (IMvigor210 dataset) (11, 12) resulting also in other tumour types.

Bravaccini et al. evaluated the expression of the immune checkpoint PD-L1 and the EMT marker vimentin expression in tumor cells, immune infiltrate and PD-L1 positive immune infiltrate through immunohistochemistry in tissue samples from resected non-metastatic NSCLC patients. A weak positive correlation was found between PD-L1 and vimentin expressions in tumor cells (r=0.25; p< 0.001) and a trend towards a shorter overall survival in patients with both PD-L1 and vimentin expression >1% (HR 1.36; 95% CI:0.96–1.93, p 0.087) suggesting that the interplay between PD-L1 and vimentin may affect the risk of tumour progression through the effect of EMT on immune evasion exerted through the regulation of PD-L1 expression (13) as a consequence of the action of TNF-α on NF-κB stimulation, which increases EMT induction by TGF-β1. NF-κB inhibition also blocks PD-L1 (13).

Although Immune checkpoint inhibitors (ICIs) have revolutionized the treatment of NSCLC, not all patients can benefit from the treatment, and secondary resistance to ICIs is widely reported. In addition to T lymphocytes, which are the major target for immunotherapies, a variety of other cells present in the tumor microenvironment (TME) act in a complex cross-talk between tumor, stromal, and immune cells. Gemelli et al. reviewed the potential role of Natural Killer (NK) cells as predictive biomarkers for immunotherapy response and putative targets to overcome resistance in NSCLC. In physiological conditions, NKs can coordinate the anticancer response together with T cells. However, cancer cells and TME act by modulating NK functions inducing the switch toward a pro-tumor phenotype influencing the treatment response and effectiveness of ICIs. Indeed a growing amount of evidence suggests that NKs can act as predictor as well as a prognostic factor but they may also represent a, may also be a promising therapeutic strategy (14, 15).

Patients affected by advanced gastric cancer show a very poor prognosis with a median survival of less than one year (16). The introduction of immunotherapy is able to improve the overall survival but not all patients show the same benefits (17). Thus, there is the need for novel and more effective biomarkers that could be used to predict progression and response to immunotherapy. In their study Liang et al. investigated the putative role of as biomarkers of chemokine related long non coding (lnc-RNA). By using TCGA expression analysis data, they constructed a risk model including 10 chemokine-related lncRNAs that were able to predict patient survival, immune cell infiltration and immunotherapy response.

In recent years post-transcriptional mRNA modifications have emerged as one of the main mechanisms of gene regulation in eukaryotes. In particular, the methylation of adenosine at messenger RNA to form m6A is the most frequent mRNA modification. Several recent studies suggest that m6A modification play an important role in the interplay between the immune system and cancer (18). Liao et al. screened 23 m6A regulatory factors in 369 colorectal cancers. The modification patterns of m6A were correlated with the characteristics of TME cell infiltration. They identified three different m6A modification patterns related to different and biological pathways and clinical outcome, that allowed the stratification of patients into high and low score groups.

In conclusion, all the collected articles provide a deeper insight into the role of TME components and the interplay between them in pathophysiology of cancer and response to treatment. Published contributions range from focus on specific TME cell types to comprehensive bioinformatic analysis of publicly online cancer-related databases, combined with experimental models in order to provide prognostic biomarkers and risk models based on TME characteristics which could improve the clinical decision making and personalized approaches. We hope that this Research Topic will contribute to increasing the comprehension of TME network thus facilitating the application of this knowledge in clinical settings.

## Author contributions

All authors listed have made a substantial, direct, and intellectual contribution to the work and approved it for publication.

## Conflict of interest

The authors declare that the research was conducted in the absence of any commercial or financial relationships that could be construed as a potential conflict of interest.

## Publisher’s note

All claims expressed in this article are solely those of the authors and do not necessarily represent those of their affiliated organizations, or those of the publisher, the editors and the reviewers. Any product that may be evaluated in this article, or claim that may be made by its manufacturer, is not guaranteed or endorsed by the publisher.
